# *Astragalus* Polysaccharides Ameliorate Diet-Induced Gallstone Formation by Modulating Synthesis of Bile Acids and the Gut Microbiota

**DOI:** 10.3389/fphar.2021.701003

**Published:** 2021-07-01

**Authors:** Qian Zhuang, Xin Ye, Shuang Shen, Jinnian Cheng, Yan Shi, Shan Wu, Jie Xia, Min Ning, Zhixia Dong, Xinjian Wan

**Affiliations:** Digestive Endoscopic Center, Shanghai Jiao Tong University Affiliated Sixth People’s Hospital, Shanghai, China

**Keywords:** gallstones, astragalus polysaccharides, cholesterol, bile acids, enterohepatic circulation, gut microbiota

## Abstract

Cholesterol gallstone (CG) disease has relationships with several metabolic abnormalities. *Astragalus* polysaccharides (APS) have been shown to have multiple benefits against metabolic disorders. We attempted to uncover the effect and mechanism of action of APS on diet-induced CG formation in mice. Animals were fed a chow diet or lithogenic diet (LD) with or without APS supplementation. The effect of APS on CG formation was evaluated. The level of individual bile acids (BAs) in gallbladder bile and ileum were measured by liquid chromatography-tandem mass spectrometry. Real-time reverse transcription-quantitative polymerase chain reaction and western blotting were used to assess expression of the genes involved in BA metabolism and the enterohepatic circulation. Cecal contents were collected to characterize microbiota profiles. APS ameliorated LD-induced CG formation in mice. APS reduced the level of total cholesterol, bile acid hydrophobicity index and cholesterol saturation index in gallbladder bile. The protective effect of APS might result from reduced absorption of cholic acid in the intestine and increased hepatic BA synthesis. APS relieved the LD-induced activation of the intestinal farnesoid X receptor and decreased ileal expression of fibroblast growth factor 15. In the liver, expression of cytochrome P450 (*Cyp*) enzyme *Cyp7a1* and *Cyp7b1* was increased, whereas expression of adenosine triphosphate-binding cassette (*Abc*) transporters *Abcg5* and *Abcg8* was decreased by APS. APS improved the diversity of the gut microbiota and increased the relative abundance of the Bacteroidetes phylum. APS had demonstratable benefits against CG disease, which might be associated with enhanced BA synthesis and improved gut microbiota. Our results suggest that APS may be a potential strategy for the prevention of CG disease.

## Introduction

Cholelithiasis is relatively common worldwide. Cholesterol gallstone (CG) disease is the most common type of cholelithiasis (Lammert et al., 2016). Cholecystectomy is the most efficacious treatment in management of symptomatic CG disease, but results in high socioeconomic costs (Everhart and Ruhl, 2009; Lammert et al., 2016). Cholesterol, bile acids (BAs), and phospholipids are the main lipids in bile. Cholesterol hypersaturation in bile is an important prerequisite for CG formation (Lammert et al., 2016; Di Ciaula et al., 2018). In the normal physiologic state, BAs and phospholipids form mixed micelles in which hydrophobic cholesterol can be solubilized. However, under certain pathophysiologic conditions (e.g., hepatic hypersecretion of cholesterol and deficiency of BAs or phospholipids), the homeostasis of biliary cholesterol can be disturbed. Supersaturated bile of cholesterol leads to the precipitation of cholesterol crystals that accumulate and grow in the gallbladder (Di Ciaula et al., 2018).

Cholesterol, BAs, and phospholipids are secreted into the biliary tract by an elaborate network of transporters. Cholesterol is secreted by the adenosine triphosphate-binding cassette (*Abc*) transporters *Abcg5* and *Abcg8* (Lammert et al., 2016). BA secretion is mediated by bile acid export pump (*Bsep*) and multidrug resistance protein (*Mrp*) 2 (Kunst et al., 2020). *Abcb4* (also known as multidrug resistance protein 3) is a major transporter of phospholipids (Poupon et al., 2013).

BA formation is another crucial catabolic pathway for cholesterol (Jia et al., 2021). BA synthesis can be accomplished *via* two routes. The classical pathway is initiated by cytochrome P450 (*Cyp*) enzyme *Cyp7a1*. The alternative pathway is initiated by *Cyp27a1* (Wahlstrom et al., 2016). *Cyp8b1* and *Cyp7b1* are also crucial enzymes involved in BA synthesis (Wahlstrom et al., 2016). Gallbladder BAs are secreted into the small intestine following food intake. Most BAs are reabsorbed in the ileum and return to the liver *via* the portal circulation (Wahlstrom et al., 2016) in a process known as the ”enterohepatic circulation of BAs”.

BA synthesis is regulated mainly by the nuclear farnesoid X receptor (*Fxr*), which shows high expression in the liver and small intestine (Kong et al., 2012). *Fxr*-mediated induction of hepatic small heterodimer partner (*Shp*) and intestinal fibroblast growth factor 15 (*Fgf15*; *FGF19* in humans) reduce expression of the genes encoding BA synthetic enzymes (Kong et al., 2012). *Fxr* has varying affinities for different BA species. Cholic acid (CA) and deoxycholic acid (DCA) are agonists of the *Fxr* (de Aguiar Vallim et al., 2013), whereas muricholic acids (MCAs) are *Fxr* antagonists (Sayin et al., 2013).

CG disease has relationships with several metabolic abnormalities (Di Ciaula et al., 2019) and is associated with alteration of the gut microbiota (Wang et al., 2017; Grigor'eva and Romanova, 2020; Wang et al., 2020b). Several types of herbal polysaccharides can improve metabolic disorders (Wu et al., 2019; Sun et al., 2020). *Astragalus* polysaccharides (APS) are natural macromolecules extracted from *Astragalus mongholicus* Bunge, which is a commonly used traditional Chinese medicine. Several studies have reported that APS can ameliorate obesity, insulin resistance, hepatic steatosis, and hypercholesteremia in mice and cell lines (Huang et al., 2017; Ke et al., 2017; Hong et al., 2020). A recent study reported that APS had beneficial effects on high-fat diet-induced metabolic disorders in mice because it could regulate intestinal metabolism as well as gut microbial structure and function (Hong et al., 2020). However, whether APS has a beneficial effect on diet-induced CG formation is not known. Here, we aimed to uncover the effect and mechanism of action of APS on lithogenic diet (LD)-induced CG formation.

## Materials and Methods

### Ethical Approval of the Study Protocol

The study protocol was approved by the Animal Care and Use Committee of Affiliated Sixth People’s Hospital within Shanghai Jiao Tong University (Shanghai, China).

### Animals and Treatment

After 1 week of acclimatization, 40 male C57BL/6J mice (aged 6 weeks) were divided randomly into four groups (10 mice in each group and five mice in each cage): Chow; LD; LD with low-dose APS (2%; AL group); LD with high-dose APS (8%; AH group).

The chow diet was LAD 0011, and the LD was TP 28900, both of which were from Trophic Diet (Nantong, China). The LD contained 15% fat, 1.25% cholesterol, and 0.5% CA with and without APS supplementation. Mice were fed these diets for 6 weeks. APS was purchased from Ci Yuan Biotechnology (Shanxi, China) and the purity of polysaccharides from *A. mongholicus* was 98%. Mice were housed in standard cages at 20–24°C with 12-h light–dark cycles and *ad libitum* access to food and water. At the end of experimentation, mice were killed after a 12-h fast.

### Serum and Bile Analyses and Cholesterol Saturation Index Calculation

Levels of metabolites in serum and bile were measured by enzymatic assays using a microplate reader (BioTek, Winooski, VT, United States ). Levels of total cholesterol (TC) and triglycerides (TG) in serum and levels of TC and BAs in gallbladder bile were measured using kits according to manufacturer (Jiancheng Bioengineering Institute, Nanjing, China) instructions. Phospholipid levels in gallbladder bile were measured according to kit instructions (Wako Pure Chemicals, Osaka, Japan). The CSI of gallbladder bile was calculated using the following equation: actual molar percentage of TC in bile/highest concentration of soluble TC at a given bile molarity in the Carey table (Carey, 1978).

### Analyses of Bile Acids Species in Bile and the Ileum

Individual BA levels in gallbladder bile and the ileum were measured by negative electrospray liquid chromatography-tandem mass spectrometry (LC-MS/MS) in multiple-reaction-monitoring mode on an Acquity ultra-high-pressure liquid chromatography (UPLC) system (Waters, Milford, MA, United States ). Thirty-eight BA standards were weighed accurately and prepared as standard solutions through serial dilution using methanol. UPLC separations were undertaken on an Acquity UPLC BEH C18 column (internal dimensions: 2.1 mm × 100 mm, 1.7 μm; Waters). The temperature of the column was set at 40°C. The injection volume of the sample was 5 μL. Eluents consisted of 0.01% formic acid in water (eluent A) and acetonitrile (eluent B). The flow rate was set at 0.25 ml/min. A 38 min elution gradient was used: 0–4 min, 25% B; 4–9 min, 25–30% B; 9–14 min, 30–36% B; 14–18 min, 36–38% B; 18–24 min, 38–50% B; 24–32 min, 50–75% B; 32–35 min, 75–100% B; 35–38 min, and 100–25% B.

For gallbladder bile, a 10 µL bile sample was mixed with 500 μL of methanol (−20°C), vortex-mixed with oscillation for 1 min, and then centrifuged (12,000 × *g*, 10 min, 4°C). Twenty microliters of supernatant was transferred to a clean tube and diluted 2000-fold with methanol. After dilution, 300 μL of supernatant was ready for LC-MS/MS. The Hydrophobicity Index (HI) of gallbladder bile were calculated as reported previously (Heuman, 1989; Posa, 2014).

For ileum samples, the ileum was flushed with physiologic (0.9%) saline and soaked with gauze. Ileum tissue was weighed precisely (∼50 mg), homogenized in 1 ml of methanol (−20°C), and centrifuged (12,000 × *g*, 10 min, 4°C). One-hundred microliters of supernatant was mixed with 900 μL of methanol, vortex-mixed with oscillation for 30 s, and passed through a 0.22-μm filter membrane. After centrifugation, the supernatant was ready for LC-MS/MS.

### Histology of Liver Tissue

Liver sections were fixed in 4% neutral-buffered formaldehyde, embedded in paraffin, cut into slices (thickness, 4 μm), and then stained with hematoxylin and eosin (H&E). To determine lipid-droplet accumulation in the liver, Oil Red O (Servicebio, Wuhan, China) staining of frozen liver sections was undertaken. The corresponding positively stained area was quantified with Image-Pro Plus v6.0.0 (Media Cybernetics, Rockville, MD, United States) and results are expressed as a percentage of the total area of a high-power field.

### Analyses of Hepatic Lipids

Hepatic lipids were extracted using the Folch method (Huang et al., 2019). Briefly, liver tissues were homogenized in a mixture of chloroform:methanol (2:1; *v/v*), followed by a series of dispersion, agitation, centrifugation, and resuspension steps. Levels of TC and TG were measured with assay kits following manufacturer instructions.

### Real-Time Reverse Transcription-Quantitative Polymerase Chain Reaction

Total RNA in liver and ileum tissues was isolated using a tissue RNA purification kit (EZBioscience, Roseville, MN, United States). The total RNA of each sample was quantified by a spectrophotometer (NanoDrop™ 2000C; Thermo Fisher, Waltham, MA, United States). Complementary (c)DNA synthesis was done using HiScript™ III RT SuperMix for qPCR (Vazyme, Nanjing, China). RT-qPCR primers were designed and synthesized (Sangon Biotech, Shanghai, China) and the sequences are listed in Table 1. RT-qPCR was undertaken using AceQ Universal SYBR® qPCR Master Mix (Vazyme) on an ABI QuantStudio seven Flex RT-PCR system (Applied Biosystems, Foster City, CA, United States ). The values of the target genes were normalized to that of glyceraldehyde 3-phosphate dehydrogenase (*Gapdh*) and the 2^−ΔΔCt^ method was used to determine relative expression of target genes.

**TABLE 1:** Primer sequences for quantitative real-time PCR analysis.

Gene	Forward primer	Reverse primer
*Cyp7b1*	GGA​GCC​ACG​ACC​CTA​GAT​G	TGC​CAA​GAT​AAG​GAA​GCC​AAC
*Cyp7a1*	AGC​AGC​CTC​TGA​AGA​AGT​GAA​TGG	AGA​GCC​GCA​GAG​CCT​CCT​TG
*Cyp8b1*	ACA​CCA​AGG​ACA​AGC​AGC​AAG​AC	TGG​CTC​ACT​TCC​ACC​CAC​TCC
*Cyp27a1*	ACA​CGG​ATG​CCT​TAA​ACG​AGG	GCA​GCC​AAT​CCT​TTT​CTC​AAA​C
*β-klotho*	TGT​TCT​GCT​GCG​AGC​TGT​TAC	CCG​GAC​TCA​CGT​ACT​GTT​TTT
*Mrp2*	GTG​TGG​ATT​CCC​TTG​GGC​TTT	CAC​AAC​GAA​CAC​CTG​CTT​GG
*Mrp3*	CTG​GGT​CCC​CTG​CAT​CTA​C	GCC​GTC​TTG​AGC​CTG​GAT​AAC
*Mrp4*	GGC​ACT​CCG​GTT​AAG​TAA​CTC	TGT​CAC​TTG​GTC​GAA​TTT​GTT​CA
*Oatp1a1*	GTG​CAT​ACC​TAG​CCA​AAT​CAC​T	CCA​GGC​CCA​TAA​CCA​CAC​ATC
*Oatp1a4*	GCT​TTT​CCA​AGA​TCA​AGG​CAT​TT	CGT​GGG​GAT​ACC​GAA​TTG​TCT
*Oatp1b2*	AGA​TCA​GAG​AAG​ACA​AGG​CAC​T	CTT​TGG​TCG​GTG​TAG​CTT​GG
*Bsep*	TCT​GAC​TCA​GTG​ATT​CTT​CGC​A	CCC​ATA​AAC​ATC​AGC​CAG​TTG​T
*Asbt*	GTC​TGT​CCC​CCA​AAT​GCA​ACT	CAC​CCC​ATA​GAA​AAC​ATC​ACC​A
*Ntcp*	CAA​ACC​TCA​GAA​GGA​CCA​AAC​A	GTA​GGA​GGA​TTA​TTC​CCG​TTG​TG
*Ostα*	AGG​CAG​GAC​TCA​TAT​CAA​ACT​TG	TGA​GGG​CTA​TGT​CCA​CTG​GG
*Ostβ*	AGA​TGC​GGC​TCC​TTG​GAA​TTA	TGG​CTG​CTT​CTT​TCG​ATT​TCT​G
*Fgf15*	ATG​GCG​AGA​AAG​TGG​AAC​GG	CTG​ACA​CAG​ACT​GGG​ATT​GCT
*Fgfr4*	GCT​CGG​AGG​TAG​AGG​TCT​TGT	CCA​CGC​TGA​CTG​GTA​GGA​A
*Abcb4*	CAG​CGA​GAA​ACG​GAA​CAG​CA	TCA​GAG​TAT​CGG​AAC​AGT​GTC​A
*Fxr*	GCT​TGA​TGT​GCT​ACA​AAA​GCT​G	CGT​GGT​GAT​GGT​TGA​ATG​TCC
*Shp*	TGG​GTC​CCA​AGG​AGT​ATG​C	GCT​CCA​AGA​CTT​CAC​ACA​GTG
*Abcg5*	AGG​GCC​TCA​CAT​CAA​CAG​AG	GCT​GAC​GCT​GTA​GGA​CAC​AT
*Abcg8*	CTG​TGG​AAT​GGG​ACT​GTA​CTT​C	GTT​GGA​CTG​ACC​ACT​GTA​GGT
*Gapdh*	AGG​TCG​GTG​TGA​ACG​GAT​TTG	TGT​AGA​CCA​TGT​AGT​TGA​GGT​CA

*Cyp7b1*, cytochrome P450 7b1; *Cyp7a1*,cytochrome P450 7a1; *Cyp8b1*, cytochrome P450 8b1; *Cyp27a1*, cytochrome P450 27a1; *β-klotho*, beta-klotho; *Mrp2*, multidrug resistance protein 2; *Mrp3*, multidrug resistance protein 3; *Mrp4*, multidrug resistance protein 4; *Oatp1a1*, organic anion-transporting polypeptide 1a1; *Oatp1a4*, organic anion-transporting polypeptide 1a4; *Oatp1b2*, organic anion-transporting polypeptide 1b2; *Bsep*, bile acid export pump; *Asbt*, apical sodium dependent bile acid transporter; *Ntcp*, Na/taurocholate cotransporting polypeptide; *Ostα*, organic solute transporter alpha; *Ostβ*, organic solute transporter beta; *Fgf15*, fibroblast growth factor 15; *Fgfr4*, fibroblast growth factor receptor 4; *Abcb4*, ATP-binding cassette; subfamily B; member 4; *Fxr*, bile acid receptor; Shp, nuclear receptor subfamily 0 group B member 2; *Abcg5*, ATP-binding cassette subfamily G member 5; *Abcg8*, ATP-binding cassette subfamily G member 8; *Gapdh*, glyceraldehyde-3-phosphate dehydrogenase.

### Western Blotting

Ileum and liver samples were homogenized in RIPA lysis buffer (Beyotime Institute of Biotechnology, Shanghai, China) containing protease and a phosphatase inhibitor cocktail (Beyotime Institute of Biotechnology). The protein extract was supplied with loading buffer (EpiZyme, Shanghai, China) and denatured by boiling at 100°C for 10 min. Equal amounts of total cellular proteins (40 μg) were separated by sodium dodecyl sulfate–polyacrylamide gel electrophoresis using 10% gels and transferred to Immobilon-P Transfer Membranes (Millipore, Tullagreen, Ireland). The latter were blocked with 5% nonfat milk in 0.1% TBS-Tween 20 for 1 h, incubated with primary antibodies overnight at 4°C, and then incubated with horseradish peroxidase-linked secondary antibody (catalog number, 7,074; Cell Signaling Technology, Danvers, MA, United States ). Bands were visualized using SuperSignal West Pico Chemiluminescent Substrate (Thermo Scientific) with a ChemiDoc MP imaging system (Bio-Rad Laboratories, Hercules, CA, United States).

*Gapdh* was included as a loading control. The antibodies used (which were all raised in rabbits and the dilution was 1:1,000) were: anti-*Gapdh* (catalog number, 5174S; Cell Signaling Technology), anti-*Fgf15* (ab229630; Abcam, Cambridge, United Kingdom), anti-*Cyp7a1* (ab65596; Abcam), anti-*Abcg8* (DF6673; Affinity, Changzhou, China), anti-*Abcg5* (27722-1-AP; Proteintech, Rosemont, IL, United States ), and anti-*Cyp7b1* (24889-1-AP; Affinity).

### Preparation of 16S rRNA Gene Libraries, and Analyses of Sequencing and Diversity

16S rRNA-sequencing of cecal contents was carried out. Briefly, total genomic DNA from samples was extracted using the cetyltrimethylammonium-bromide method. 16S rRNA genes of distinct regions (V3–V4) were amplified using specific primers. The mixture of PCR products was purified with a gel extraction kit (Qiagen, Hilden, Germany). Sequencing libraries were generated using the TruSeq® DNA PCR-Free Sample Preparation kit (Illumina, San Diego, CA, United States) following manufacturer instructions, and index codes were added. A Qubit@2.0 fluorometer (Thermo Fisher) and bioanalyzer (2,100 system; Agilent Technologies, Santa Clara, CA, United States ) were used to assess the quality of the library. The latter was sequenced on a NovaSeq platform (Illumina), and 250-bp paired-end reads were generated.

Sequencing was done using Uparse v7.0.1001 (http://drive5.com/uparse/) (Edgar, 2013). Operational taxonomic unit (OTU) analyses enabled clustering of sequences at a similarity level of 97%. Representative sequences for each OTU were screened for further annotation. The Silva Database (www.arb-silva.de/) (Quast et al., 2013) was used based on the Mothur algorithm to annotate taxonomic information. Information on OTU abundance was normalized using a standard sequence number corresponding to the sample with the fewest sequences. Alpha diversity was employed to analyze the complexity of species diversity and was calculated with QIIME 1.7.0 (http://qiime.org/). Beta-diversity analyses were used to evaluate differences in samples with regard to species complexity. Beta diversity on weighted UniFrac was calculated by QIIME 1.9.1. Principal coordinate analysis (PCoA) was undertaken to obtain principal coordinates and visualize complex, multidimensional data.

### Statistical Analyses

Data are the mean ± standard deviation (SD). Appropriate statistical analyses were applied depending on data distribution. For data that showed a normal distribution, the two-tailed Student’s *t*-test was used between two groups, and one-way analysis of variance followed by Tukey’s test for multiple comparisons was used between three or four groups. For datasets with a skewed distribution, the Mann–Whitney test was used between two groups, and the Kruskal–Wallis test followed by Dunn’s test for multiple comparisons was done between three or four groups. Statistical analyses were undertaken using Prism 8 (www.graphpad.com). Correlations between the level of BAs in the ileum and relative abundance of the microbiome were carried out using Spearman’s correlation analysis followed by *p*-value correction of the false discovery rate, which was visualized using R 3.6.2 (R Institute for Statistical Computing, Vienna, Austria; www.r-project.org/). *p* < 0.05 was considered significant.

## Results

### *Astragalus* polysaccharides Ameliorated Lithogenic Diet-Induced Cholesterol Gallstone Formation in Mice

**FIGURE 1:**
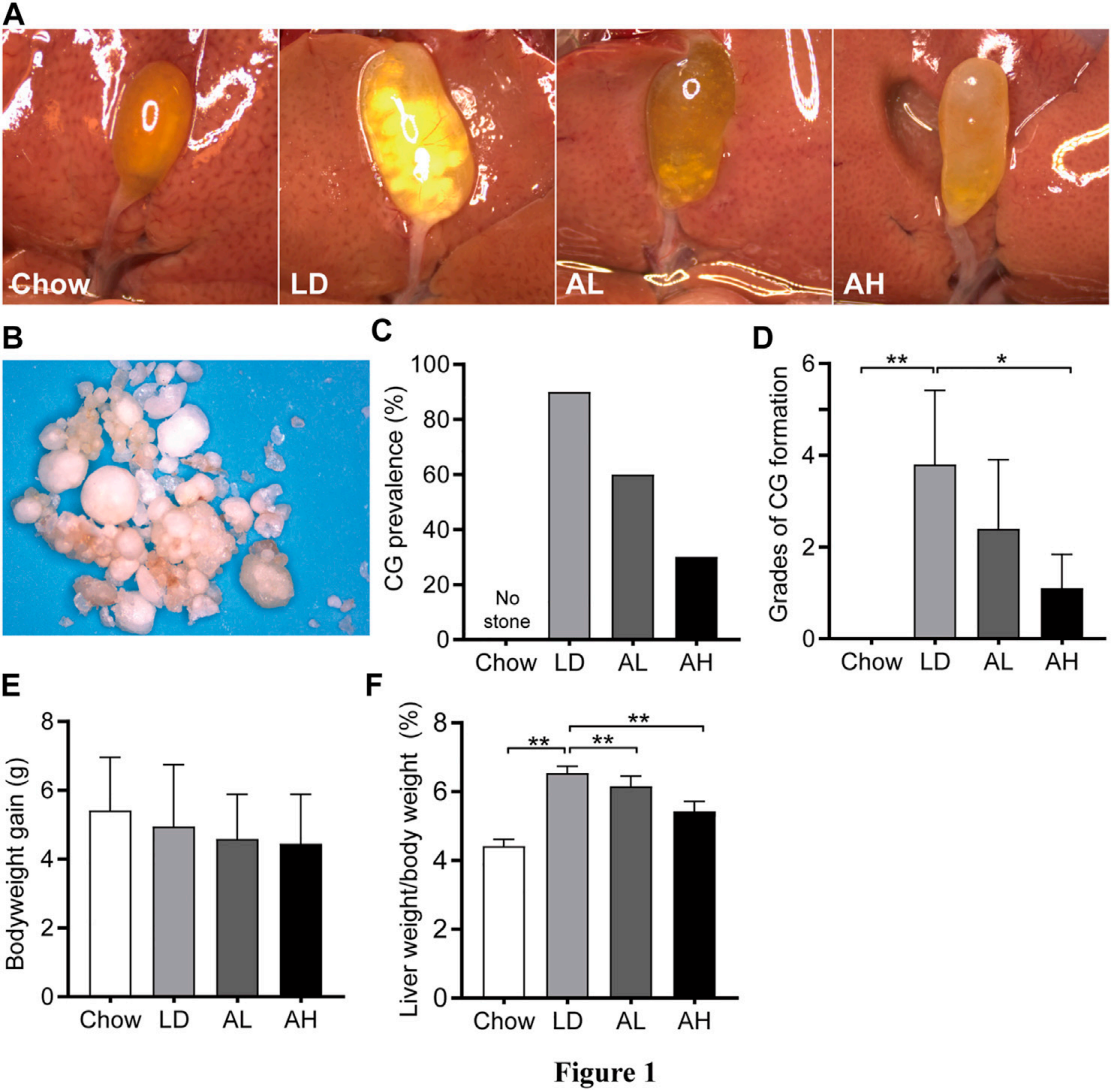
Bodyweight, and liver weight, and cholesterol gallstone (CG) prevalence. **(A)** Representative gallbladders per group. **(B)** CGs from the LD group. **(C)** CG prevalence. **(D)** Grades of CG formation. **(E)** Bodyweight gain. **(F)** Ratio of liver weight-to-bodyweight. Data are the mean ± SD (*n* = 10). *p < 0.05, **p < 0.01. Chow, chow diet; LD, lithogenic diet; AL, low-dose APS; AH, high-dose APS.

Mice in the chow group did not have CG formation and had clear gallbladders. Ninety percent of mice in the LD group had CGs and ”cloudy” gallbladders (Figures 1A,C). The color and morphology of CGs in mice are shown in Figure 1B. With APS supplementation, the prevalence of CG formation in the AL group and AH group was 60 and 30%, respectively, (Figures 1A,C). We graded CGs according to the method described by Akiyoshi and colleagues (Akiyoshi et al., 1986). The grade of CG formation in mice fed the LD was significantly higher than that of chow diet-fed mice, and was decreased significantly upon APS supplementation (Figure 1D). Bodyweight gain was not significantly different among the groups (Figure 1E). Compared with chow diet-fed mice, the ratio of liver weight-to-bodyweight was significantly higher in mice fed the LD, and APS could reverse these changes partially (Figure 1F).

### *Astragalus* polysaccharides Altered Biliary Composition and the Cholesterol Saturation Index in Mice fed the Lithogenic Diet

**FIGURE 2:**
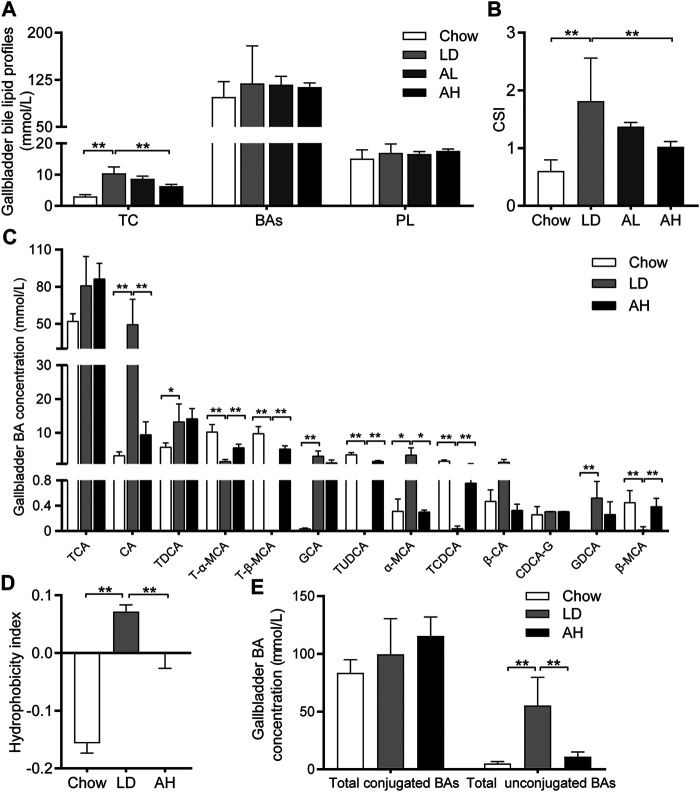
Biliary Cholesterol Saturation Index (CSI) and Hydrophobicity Index (HI) of bile acids (BAs) in gallbladder bile. **(A)** Total cholesterol (TC), BAs and phospholipids (PL) (*n* = 8–10). **(B)** CSI (n = 8–10). **(C)** Analysis of BA species (*n* = 6). Top-13 most abundant BA species are shown. **(D)** HI of BAs (*n* = 6). **(E)** Total conjugated BAs and unconjugated BAs (*n* = 6). Data are the mean ± SD, *p < 0.05, **p < 0.01. Chow, chow diet; LD, lithogenic diet; AL, low-dose APS; AH, high-dose APS.

We evaluated lipid profiles in gallbladder bile to understand the mechanism involved in the reduction of CG formation by APS. Compared with that in the chow group, the TC level was increased significantly in the gallbladder bile of mice fed the LD, whereas APS decreased the TC level significantly (Figure 2A). However, total BAs and phospholipids in gallbladder bile was not altered significantly by APS supplementation (Figure 2A). The CSI in the LD group was higher than that in the chow group, and APS supplementation reduced it (Figure 2B).

The solubility and hydrophobicity of individual BAs were different. The rank order from hydrophobic to hydrophilic was: lithocholic acid (LCA) > DCA > chenodeoxycholic acid (CDCA) > CA > ursodeoxycholic acid (UDCA) > MCA (de Aguiar Vallim et al., 2013). We employed BA-targeted metabolomics analysis to determine the changes in individual BAs in gallbladder bile. Compared with that in the chow group, the level of CA, TDCA, GCA, α-MCA, and GDCA in gallbladder bile was increased significantly in the LD group. APS administration reduced the level of CA and *α*-MCA significantly, and increased the level of T-α-MCA, T-β-MCA, TUDCA, TCDCA, and *β*-MCA, in gallbladder bile (Figure 2C). The percentage of BAs in gallbladder bile is shown in Supplementary Figure S1. The HI of gallbladder-bile BAs in the LD group was significantly higher than that in the chow group, but APS supplementation reduced the HI (Figure 2D). Principal component analysis (PCA) of BAs in gallbladder bile showed clear separation among groups (Supplementary Figure S2, left). The level of total unconjugated BAs in bile was increased significantly in the LD group, whereas APS reversed these changes (Figure 2E). However, the level of total conjugated BAs in bile was not significantly different among the three groups (Figure 2E). These results indicated that APS could reduce the TC level and increase the level of hydrophilic BA, which might attenuate cholesterol hypersaturation in gallbladder bile.

### *Astragalus* polysaccharides Ameliorated Lithogenic Diet-Induced Metabolic Disorders

**FIGURE 3:**
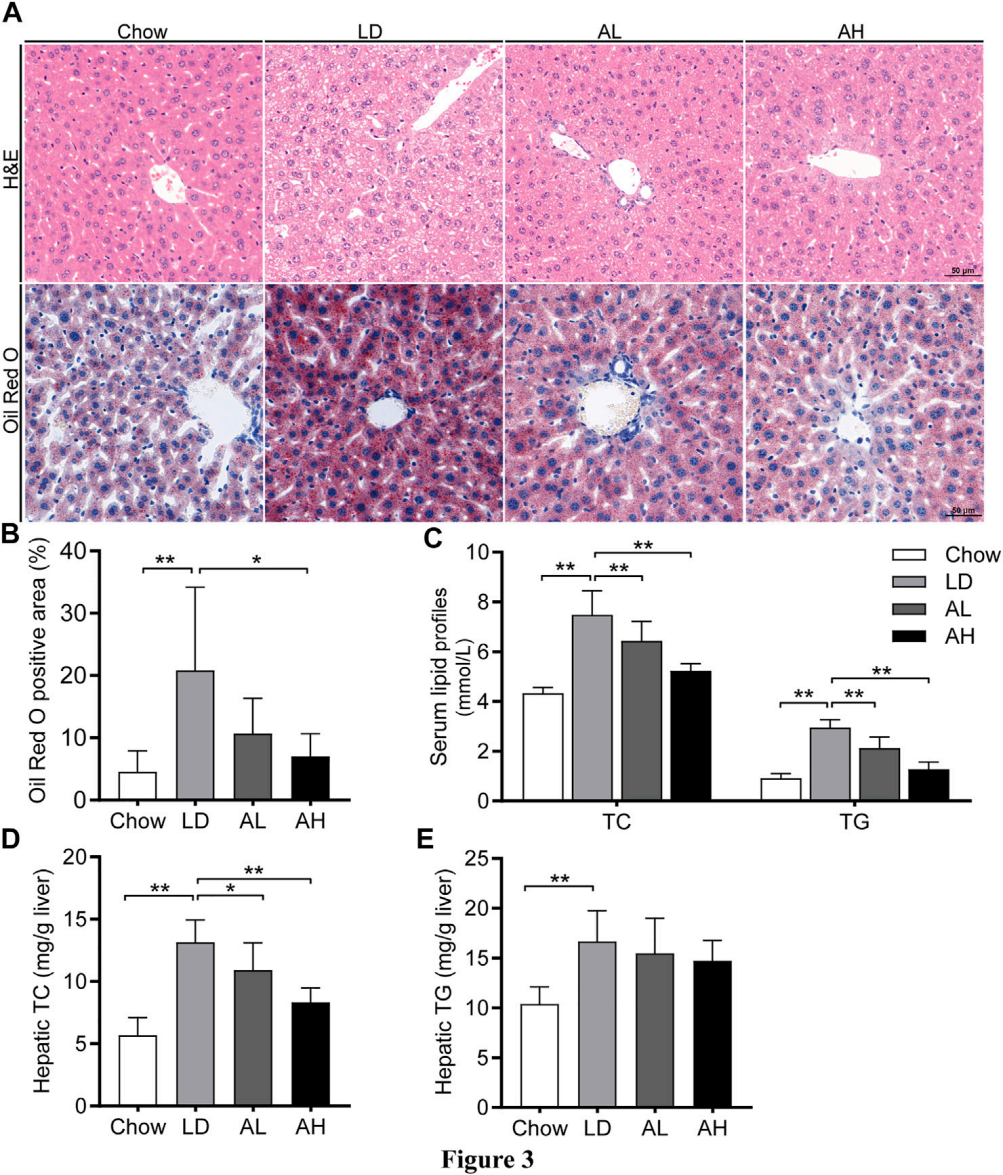
APS attenuated LD-induced metabolic disorders. **(A)** Representative images of H&E and Oil Red O staining per group (scale bar: 50 μm). **(B)** Percentage of Oil Red O positive area. **(C)** Serum levels of total cholesterol (TC) and triglycerides (TG). **(D)** Hepatic level of TC. **(E)** Hepatic level of TG. Data are the mean ± SD (*n* = 10). *p < 0.05, **p < 0.01. Chow, chow diet; LD, lithogenic diet; AL, low-dose APS; AH, high-dose APS.

LD administration resulted in significant hepatic steatosis (as revealed by H&E and Oil Red O staining), whereas APS supplementation improved hepatic steatosis (Figures 3A,B). APS significantly reduced the hepatic TC level and serum level of TC and TG, which were increased by LD consumption (Figures 3C,D). The hepatic level of TG was not reduced significantly by APS (Figure 3E). These results indicated that APS could ameliorate LD-induced metabolic disorders in mice.

### *Astragalus* polysaccharides Reduced the Cholic Acid in the Ileum and Downregulated Expression of the *Fxr*–*Fgf15* Axis

**FIGURE 4:**
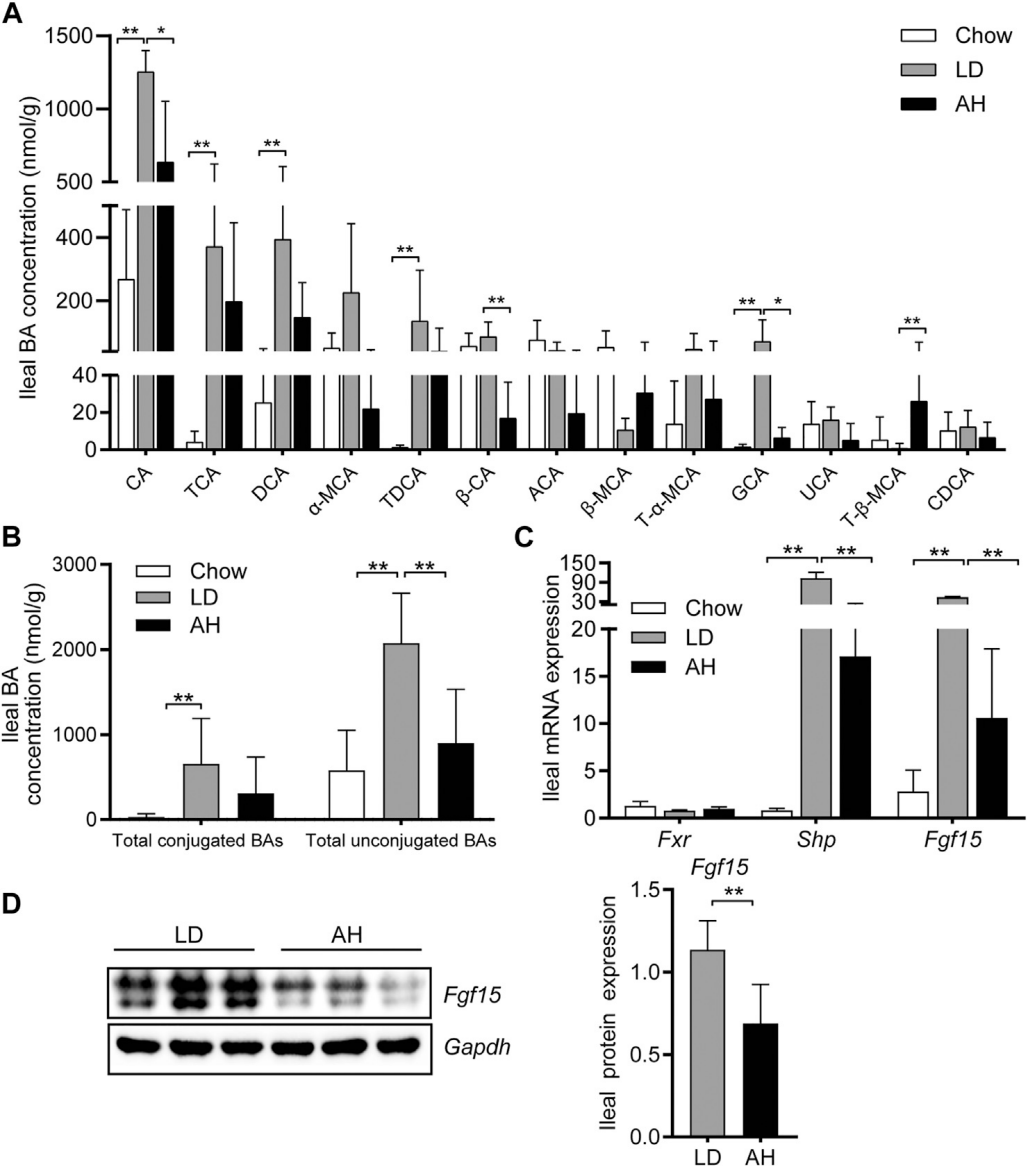
Bile acid (BA) species in the ileum and *Fxr*–*Fgf15* axis. **(A)** Analyses of BA species (*n* = 10). Top-13 most abundant BA species are shown. **(B)** Total conjugated BAs and unconjugated BAs (*n* = 10). **(C)** Ileal mRNA expression of *Fxr*, *Shp*, and *Fgf15* (*n* = 8). **(D)** Ileal expression (left panel) and quantification (right panel) of *Fgf15* protein (*n* = 6). Data are the mean ± SD. *p < 0.05, **p < 0.01. Chow, chow diet; LD, lithogenic diet; AH, high-dose APS.

We analyzed the individual BA species in the ileum (where most BAs are reabsorbed) to explore the influence of APS on BA metabolism in the intestine (Huang et al., 2019; Jia et al., 2021). APS significantly reduced the CA level in the ileum, which was increased by LD consumption (Figure 4A). After APS supplementation, the level of T-β-MCA was increased significantly (Sayin et al., 2013) (Figure 4A). The level of total conjugated BAs and total unconjugated BAs was increased in mice fed the LD, whereas APS reduced the level of total unconjugated BAs significantly (Figure 4B).

Next, we analyzed expression of *Fgf15* and *Shp* in the ileum (both of which are target genes of *Fxr*) (Kong et al., 2012). APS decreased the mRNA expression of *Fgf15* and *Shp* in the ileum significantly, which was increased significantly by LD consumption (Figure 4C). Western blotting further confirmed the reduced protein expression of *Fgf15* in APS-treated mice (Figure 4D).

### *Astragalus* polysaccharides Decreased the Canalicular Efflux of Cholesterol and Enhanced Bile Acids Synthesis

**FIGURE 5:**
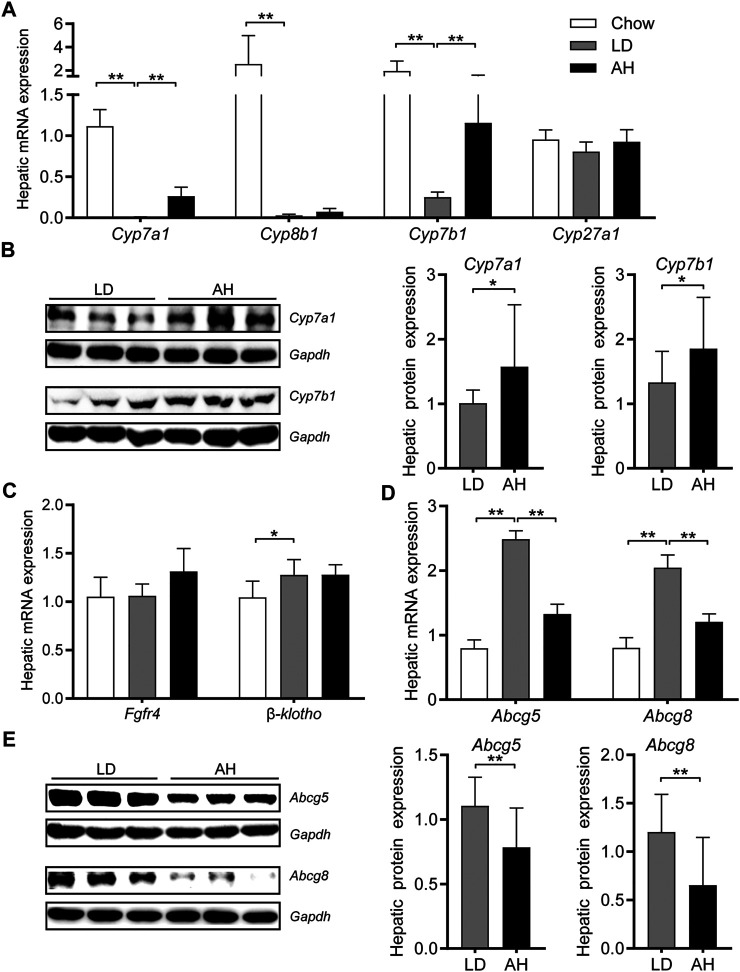
Expression of the genes involved in cholesterol transport and bile-acid synthesis in the liver. **(A)** mRNA expression of *Cyp7a1*, *Cyp8b1*, *Cyp7b1*, and *Cyp27a1* (*n* = 8). **(B)** Protein expression (left panel) and quantification (middle and right panel) of *Cyp7a1* and *Cyp7b1* (*n* = 6). **(C)** mRNA expression of *Fgfr4* and *β-klotho* (*n* = 8). **(D)** mRNA expression of *Abcg5* and *Abcg8* (*n* = 8). **(E)** Protein expression (left panel) and quantification (middle and right panel) of *Abcg5* and *Abcg8* (*n* = 6). Data are the mean ± SD. *p < 0.05, **p < 0.01. Chow, chow diet; LD, lithogenic diet; AH, high-dose APS.

Consistent with the activation of intestinal *Fxr*, we found that hepatic mRNA expression of *Cyp7a1*, *Cyp8b1*, and *Cyp7b1* was decreased significantly in mice fed the LD (Figure 5A). APS significantly increased hepatic mRNA expression of *Cyp7a1* and *Cyp7b1* (Figure 5A). Western blotting confirmed the increased protein level of *Cyp7a1* and *Cyp7b1* in APS-treated mice (Figure 5B). Consistent with the increased *de novo* synthesis of BAs from cholesterol, the hepatic level of cholesterol was reduced (Figure 3D). Compared with the LD group, expression of *Fgf* receptor 4 (*Fgfr4*) and its coreceptor *β-klotho* was unchanged in APS-treated mice (Figure 5C). Hepatic expression of *Fxr* and *Shp* was unchanged (Supplementary Figure S3D), which suggested that hepatic *Fxr* might not account for the increased expression of *Cyp7a1* and *Cyp7b1* in APS-treated mice. APS reduced mRNA and protein expression of *Abcg5* and *Abcg8* significantly, which were increased by LD consumption (Figures 5D,E). This finding was consistent with the reduced cholesterol level in gallbladder bile (Figure 2A). Taken together, these results indicated that increased expression of *Cyp7a1* and *Cyp7b1* might have resulted from the inhibition of intestinal *Fxr* and decreased *Fgf15* expression in the ileum.

### *Astragalus* polysaccharides Changed Expression of the Genes Involved in Bile Acids Transport in the Liver and Ileum

Uptake and efflux of BAs are mediated by a series of efficient BA transporters in the liver. Sodium taurocholate cotransporting polypeptide (*Ntcp*) and organic anion-transporting polypeptides (*Oatps*) are responsible for transporting BAs from portal blood into hepatocytes (Kunst et al., 2020). mRNA expression of *Oatp1a1* and *Oatp1a4* was decreased significantly and increased in mice fed the LD, respectively, whereas APS reversed these changes (Supplementary Figure S3A). mRNA expression of *Oatp1b2* and *Ntcp* did not change significantly in any group (Supplementary Figure S3A). For sinusoidal uptake transporters, increased mRNA expression of *Mrp3* and *Mrp4* in the LD group was decreased significantly by APS supplementation (Supplementary Figure S3A). *Bsep* and *Mrp2* pump bile salts out of hepatocytes into primary bile (Kunst et al., 2020). mRNA expression of *Bsep* was increased significantly in mice fed the LD, but APS had little effect on mRNA expression of *Bsep* and *Mrp2* (Supplementary Figure S3A). mRNA expression of *Abcb4*—a major phospholipid transporter (Poupon et al., 2013)—was not affected significantly (Supplementary Figure S3C).

Apical sodium-dependent bile acid transporter (*Asbt*) actively reabsorbs BAs in the distal ileum, and the reabsorbed BAs are then transported into the portal circulation by organic solute transporter α/β (*Ostα/β*) (Ticho et al., 2019). LD consumption decreased expression of *Asbt* mRNA and *Ostβ* mRNA in the ileum significantly, which might have been caused by activation of intestinal *Fxr* (Ticho et al., 2019), whereas APS normalized these changes partly (Supplementary Figure S3B).

### *Astragalus* polysaccharides Reversed Lithogenic Diet-Induced Gut Dysbiosis in Mice

**FIGURE 6:**
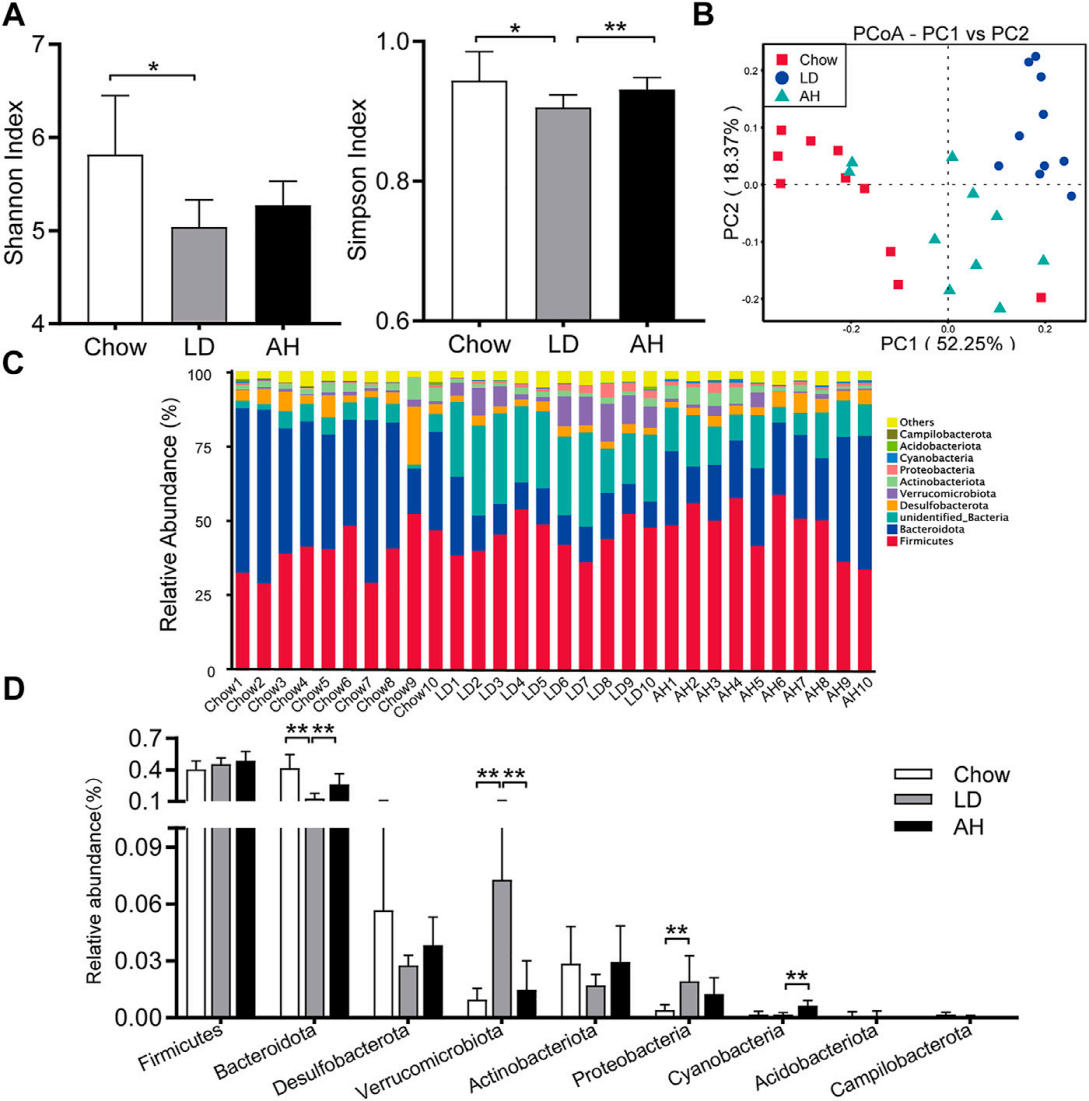
APS changed the composition of the gut microbiota. **(A)** Shannon Index and Simpson Index. **(B)** Weighted Unifrac principal coordinate analysis (PCoA). **(C, D)** Multigroup difference analysis of the top-9 most abundant phyla. Data are the mean ± SD (*n* = 10). *p < 0.05, **p < 0.01. Chow, chow diet; LD, lithogenic diet; AH, high-dose APS.

We further explored the influence of APS on the gut microbiota by 16S rRNA-sequencing. The Shannon Index and Simpson Index were reduced significantly in the LD group, and the Simpson Index was increased significantly by APS supplementation, which suggested that APS could improve the diversity of the gut microbiota (Figure 6A). Weighted UniFrac PCoA showed clear separation among groups (Figure 6B). Taxonomic profiling of the top-nine most abundant phyla showed that LD consumption significantly increased the relative abundance of the phyla Verrucomicrobia, Proteobacteria and Cyanobacteria and reduced the relative abundance of the phylum Bacteroidetes, whereas APS reversed the changes in the abundance of Bacteroidetes and Verrucomicrobia (Figures 6C,D). Considering the intestinal crosstalk between BAs and microbiota, we undertook Spearman’s correlation analysis between BAs in the ileum and the relative abundance of bacterial genera. In general, the abundance of *[Eubacterium]_fissicatena_group*, *Intestinimonas*, and *Negativibacillus* genera was correlated negatively with the level of β-MCA and T-β-MCA, whereas the abundance of the *Muribaculum* genus was correlated positively with the T-β-MCA level (Supplementary Figure S4).

## Discussion

We found that APS could ameliorate LD-induced CG formation in mice. APS supplementation decreased the level of TC, the HI of BAs and CSI in bile. The protective effect of APS might result from reduced absorption or reabsorption of CA in the intestine and increased BA synthesis in the liver. Increased expression of *Cyp7a1* and *Cyp7b1* might have resulted from inhibition of intestinal *Fxr* and decreased expression of *Fgf15* in the ileum. APS also improved LD-induced gut dysbiosis.

CG disease has relationships with several metabolic abnormalities: obesity, type-2 diabetes mellitus, atherosclerosis, and nonalcoholic fatty liver disease (Lammert et al., 2016; Di Ciaula et al., 2019). Recent studies have indicated that APS can ameliorate obesity, insulin resistance, hepatic steatosis, and hypercholesteremia in mice and cell lines (Huang et al., 2017; Ke et al., 2017; Hong et al., 2020). We found that APS could ameliorate LD-induced CG formation in mice. In addition, APS improved LD-induced hepatic steatosis and hypercholesterolemia.

Studies have shown that dietary fiber can protect against CG formation in prairie dogs, and this effect was associated with a decrease in the cholesterol level in bile (Schwesinger et al., 1999). Reduced reabsorption of BAs in the ileum is considered a major mechanism that explains the cholesterol-reducing effects of dietary fibers (Naumann et al., 2020). Several studies have reported that the viscous and adsorptive effects of dietary fibers can reduce BA reabsorption in the intestine (Naumann et al., 2018; Naumann et al., 2019; Naumann et al., 2020). In the intestine, BAs are absorbed in two ways: active and passive (Ticho et al., 2019). Active absorption relies on transepithelial transporters located on apical and basolateral sides, such as *Asbt* and *Ostα/β* in the ileum (Ticho et al., 2019). BAs can also diffuse passively through membranes, and passive absorption can occur in all regions of the small and large intestines (Ticho et al., 2019). We found that APS could inhibit BA absorption and reduce the CA level in the ileum, and that the level of T-β-MCA was increased. APS decreased the LD-induced expression of *Fgf15*, a target gene of *Fxr* (Kong et al., 2012). *Fgf15* reaches the liver through portal blood, where it binds to the *Fgfr4/β-klotho* heterodimer complex to inhibit BA synthesis in the liver (Wahlstrom et al., 2016).

Cholesterol hypersaturation is a prerequisite for CG formation, and excessive hepatic secretion of cholesterol is a cause of supersaturated bile (Lammert et al., 2016). Hydrophilic BAs such as β-MCA and UDCA can act as biliary cholesterol-desaturating agents for the prevention and dissolution of CGs (Jazrawi et al., 1992; Wang and Tazuma, 2002). In the present study, in accordance with the relieved activation of intestinal *Fxr* and decreased *Fgf15* expression in the ileum, hepatic expression of *Cyp7a1*—the rate-limiting enzyme for BA synthesis (Wahlstrom et al., 2016)—and *Cyp7b1* was increased. Enhanced BA synthesis can increase cholesterol depletion. As a result, the cholesterol level in the bile and liver was reduced, and expression of *Abcg5* and *Abcg8* [which are responsible for biliary cholesterol secretion (Lammert et al., 2016)] was also decreased by APS. More hydrophilic BAs, such as T-α-MCA, T-β-MCA and TUDCA, were synthesized in mice, which decreased the HI of BAs and increased cholesterol solubility in bile. It has been reported that the biliary secretion of phospholipids and cholesterol is coupled to that of BAs under physiological conditions (Scherstén et al., 1971; Carulli et al., 1984; Gooijert et al., 2015). Several studies find that acute BAs infusion stimulates biliary secretion of phospholipids and cholesterol (Carulli et al., 1984; Gooijert et al., 2015). Whereas there is hypersaturation of the bile with cholesterol in mice fed LD, whether the correlation is altered or not still needs to be further explored. Hepatic *Shp* also regulates expression of *Cyp7a1* and *Cyp7b1* (Kong et al., 2012). However, a significant change in hepatic *Shp* expression was not observed, which suggested that hepatic *Fxr* might not account for the increased expression of *Cyp7a1* and *Cyp7b1* noted in APS-treated mice.

Recent studies have shown that CG formation is associated with alterations in the gut microbiota (Wang et al., 2017; Grigor'eva and Romanova, 2020; Wang et al., 2020b). APS is a type of prebiotic that can modulate the gut microbiome and host metabolism (Hong et al., 2020). We discovered that APS improved the diversity of the gut microbiota and increased the relative abundance of bacteria belonging to the Bacteroidetes phylum, data that are consistent with results from a study by Hong and coworkers (Hong et al., 2020).

Our study had two main limitations. First, the relative BA composition in BA pools in the humans and mice is different (de Aguiar Vallim et al., 2013). Mouse models of gallstones established with a combination of cholesterol and cholic acid are used widely (Wang et al., 2018; Munoz et al., 2019; Wang et al., 2020a), but the pathogenesis of CG formation in mice and humans is not identical. For example, humans do not ingest CA *via* the diet. When translating findings from mice to humans, these differences should be taken into account. Second, the hypotheses that lower CA absorption, enhanced BA synthesis, or microbial changes cause beneficial effects must be verified by additional mechanistic experiments, which we will do in the future.

## Conclusion

Although cholecystectomy is efficacious treatment for CG disease, it has some limitations, such as surgical complications and high socioeconomic costs. More emphasis should be given to the prevention of CG disease. We found that the herbal polysaccharide APS demonstrated benefits against CG disease, which may be associated with enhanced BA synthesis and improved gut microbiota. Our study provides preliminary findings of APS preventing CG in a murine model. Future research is required to understand the underlying mechanism and the potential relevance to the prevention of human CG disease.

## Data Availability

The data presented in the study are deposited in the NCBI BioProject repository, accession number PRJNA725334.

## References

[B1] Akiyoshi T. Uchida K. Takase H. Nomura Y. Takeuchi N. (1986). Cholesterol Gallstones in Alloxan-Diabetic Mice. J. Lipid Res. 27 (9), 915–924. 3783046

[B2] Carey M. C. (1978). Critical Tables for Calculating the Cholesterol Saturation of Native Bile. J. Lipid Res. 19 (8), 945–955. 10.1016/s0022-2275(20)40677-7 731129

[B3] Carulli N. Loria P. Bertolotti M. Ponz de Leon M. Menozzi D. Medici G. (1984). Effects of Acute Changes of Bile Acid Pool Composition on Biliary Lipid Secretion. J. Clin. Invest. 74 (2), 614–624. 10.1172/jci111459 6746909 PMC370514

[B4] de Aguiar Vallim T. Q. Tarling E. J. Edwards P. A. (2013). Pleiotropic Roles of Bile Acids in Metabolism. Cell Metab 17 (5), 657–669. 10.1016/j.cmet.2013.03.013 23602448 PMC3654004

[B5] Di Ciaula A. Wang D. Q.-H. Portincasa P. (2018). An Update on the Pathogenesis of Cholesterol Gallstone Disease. Curr. Opin. Gastroenterol. 34 (2), 71–80. 10.1097/MOG.0000000000000423 29283909 PMC8118137

[B6] Di Ciaula A. Wang D. Q.-H. Portincasa P. (2019). Cholesterol Cholelithiasis: Part of a Systemic Metabolic Disease, Prone to Primary Prevention. Expert Rev. Gastroenterol. Hepatol. 13 (2), 157–171. 10.1080/17474124.2019.1549988 30791781

[B7] Edgar R. C. (2013). UPARSE: Highly Accurate OTU Sequences from Microbial Amplicon Reads. Nat. Methods 10 (10), 996–998. 10.1038/nmeth.2604 23955772

[B8] Everhart J. E. Ruhl C. E. (2009). Burden of Digestive Diseases in the United States Part III: Liver, Biliary Tract, and Pancreas. Gastroenterology 136 (4), 1134–1144. 10.1053/j.gastro.2009.02.038 19245868

[B9] Gooijert K. E. R. Havinga R. Wolters H. Wang R. Ling V. Tazuma S. (2015). The Mechanism of Increased Biliary Lipid Secretion in Mice with Genetic Inactivation of Bile Salt export Pump. Am. J. Physiology-Gastrointestinal Liver Physiol. 308 (5), G450–G457. 10.1152/ajpgi.00391.2014 25552583

[B10] Grigor'eva I. N. Romanova T. I. (2020). Gallstone Disease and Microbiome. Microorganisms 8 (6), 835. 10.3390/microorganisms8060835 PMC7356158 32498344

[B11] Heuman D. M. (1989). Quantitative Estimation of the Hydrophilic-Hydrophobic Balance of Mixed Bile Salt Solutions. J. Lipid Res. 30 (5), 719–730. 10.1016/s0022-2275(20)38331-0 2760545

[B12] Hong Y. Li B. Zheng N. Wu G. Ma J. Tao X. (2020). Integrated Metagenomic and Metabolomic Analyses of the Effect of Astragalus Polysaccharides on Alleviating High-Fat Diet-Induced Metabolic Disorders. Front. Pharmacol. 11, 833. 10.3389/fphar.2020.00833 32587515 PMC7299173

[B13] Huang F. Zheng X. Ma X. Jiang R. Zhou W. Zhou S. (2019). Theabrownin from Pu-Erh tea Attenuates Hypercholesterolemia via Modulation of Gut Microbiota and Bile Acid Metabolism. Nat. Commun. 10 (1), 4971. 10.1038/s41467-019-12896-x 31672964 PMC6823360

[B14] Huang Y.-C. Tsay H.-J. Lu M.-K. Lin C.-H. Yeh C.-W. Liu H.-K. (2017). Astragalus Membranaceus-Polysaccharides Ameliorates Obesity, Hepatic Steatosis, Neuroinflammation and Cognition Impairment without Affecting Amyloid Deposition in Metabolically Stressed APPswe/PS1dE9 Mice. Ijms 18 (12), 2746. 10.3390/ijms18122746 PMC5751345 29258283

[B15] Jazrawi R. P. Pigozzi M. G. Galatola G. Lanzini A. Northfield T. C. (1992). Optimum Bile Acid Treatment for Rapid Gall Stone Dissolution. Gut 33 (3), 381–386. 10.1136/gut.33.3.381 1568660 PMC1373833

[B16] Jia W. Wei M. Rajani C. Zheng X. (2021). Targeting the Alternative Bile Acid Synthetic Pathway for Metabolic Diseases. Protein Cell 12 (5), 411–425. 10.1007/s13238-020-00804-9 33252713 PMC8106556

[B17] Ke B. Ke X. Wan X. Yang Y. Huang Y. Qin J. (2017). Astragalus Polysaccharides Attenuates TNF-α-Induced Insulin Resistance via Suppression of miR-721 and Activation of PPAR-γ and PI3K/AKT in 3T3-L1 Adipocytes. Am. J. Transl. Res. 9 (5), 2195–2206. 28559971 PMC5446503

[B18] Kong B. Wang L. Chiang J. Y. L. Zhang Y. Klaassen C. D. Guo G. L. (2012). Mechanism of Tissue-specific Farnesoid X Receptor in Suppressing the Expression of Genes in Bile-Acid Synthesis in Mice. Hepatology 56 (3), 1034–1043. 10.1002/hep.25740 22467244 PMC3390456

[B19] Kunst R. F. Verkade H. J. Oude Elferink R. P. J. Graaf S. F. J. (2021). Targeting the Four Pillars of Enterohepatic Bile Salt Cycling; Lessons from Genetics and Pharmacology. Hepatology 73, 2577–2585. 10.1002/hep.31651 33222321 PMC8252069

[B20] Lammert F. Gurusamy K. Ko C. W. Miquel J.-F. Méndez-Sánchez N. Portincasa P. (2016). Gallstones. Nat. Rev. Dis. Primers 2, 16024. 10.1038/nrdp.2016.24 27121416

[B21] Muñoz L. E. Boeltz S. Bilyy R. Schauer C. Mahajan A. Widulin N. (2019). Neutrophil Extracellular Traps Initiate Gallstone Formation. Immunity 51 (3), 443–450. 10.1016/j.immuni.2019.07.002 31422870

[B22] Naumann S. Haller D. Eisner P. Schweiggert-Weisz U. (2020). Mechanisms of Interactions between Bile Acids and Plant Compounds-A Review. Ijms 21 (18), 6495. 10.3390/ijms21186495 PMC7555273 32899482

[B23] Naumann S. Schweiggert-Weisz U. Bader-Mittermaier S. Haller D. Eisner P. (2018). Differentiation of Adsorptive and Viscous Effects of Dietary Fibres on Bile Acid Release by Means of *In Vitro* Digestion and Dialysis. Ijms 19 (8), 2193. 10.3390/ijms19082193 PMC6121312 30060480

[B24] Naumann S. Schweiggert-Weisz U. Haller D. Eisner P. (2019). Retention of Primary Bile Acids by Lupin Cell Wall Polysaccharides under *In Vitro* Digestion Conditions. Nutrients 11 (9), 2117. 10.3390/nu11092117 PMC6769765 31492011

[B25] Posa M. (2014). Heuman Indices of Hydrophobicity of Bile Acids and Their Comparison with a Newly Developed and Conventional Molecular Descriptors. Biochimie 97, 28–38. 10.1016/j.biochi.2013.09.010 24076126

[B26] Poupon R. Rosmorduc O. Boëlle P. Y. Chrétien Y. Corpechot C. Chazouillères O. (2013). Genotype-phenotype Relationships in the Low-Phospholipid-Associated Cholelithiasis Syndrome: a Study of 156 Consecutive Patients. Hepatology 58 (3), 1105–1110. 10.1002/hep.26424 23533021

[B27] Quast C. Pruesse E. Yilmaz P. Gerken J. Schweer T. Yarza P. (2013). The SILVA Ribosomal RNA Gene Database Project: Improved Data Processing and Web-Based Tools. Nucleic Acids Res. 41 (database issue), D590–D596. 10.1093/nar/gks1219 23193283 PMC3531112

[B28] Sayin S. I. Wahlström A. Felin J. Jäntti S. Marschall H.-U. Bamberg K. (2013). Gut Microbiota Regulates Bile Acid Metabolism by Reducing the Levels of Tauro-Beta-Muricholic Acid, a Naturally Occurring FXR Antagonist. Cel Metab. 17 (2), 225–235. 10.1016/j.cmet.2013.01.003 23395169

[B29] Scherstén T. Nilsson S. Cahlin E. Filipson M. Brodin-Persson G. (1971). Relationship between the Biliary Excretion of Bile Acids and the Excretion of Water, Lecithin, and Cholesterol in Man. Eur. J. Clin. Invest. 1 (4), 242–247. 10.1111/eci.1971.1.4.242 5549528

[B30] Schwesinger W. H. Kurtin W. E. Page C. P. Stewart R. M. Johnson R. (1999). Soluble Dietary Fiber Protects against Cholesterol Gallstone Formation. Am. J. Surg. 177 (4), 307–310. 10.1016/s0002-9610(99)00047-1 10326849

[B31] Sun S. Wang K. Sun L. Cheng B. Qiao S. Dai H. (2020). Therapeutic Manipulation of Gut Microbiota by Polysaccharides of Wolfiporia Cocos Reveals the Contribution of the Gut Fungi-Induced PGE2 to Alcoholic Hepatic Steatosis. Gut Microbes 12 (1), 1830693. 10.1080/19490976.2020.1830693 33106075 PMC7592601

[B32] Ticho A. L. Malhotra P. Dudeja P. K. Gill R. K. Alrefai W. A. (2019). Intestinal Absorption of Bile Acids in Health and Disease. Compr. Physiol. 10 (1), 21–56. 10.1002/cphy.c190007 31853951 PMC7171925

[B33] Wahlström A. Sayin S. I. Marschall H.-U. Bäckhed F. (2016). Intestinal Crosstalk between Bile Acids and Microbiota and its Impact on Host Metabolism. Cel Metab. 24 (1), 41–50. 10.1016/j.cmet.2016.05.005 27320064

[B34] Wang D. Q.-H. Tazuma S. (2002). Effect of β-muricholic Acid on the Prevention and Dissolution of Cholesterol Gallstones in C57L/J Mice. J. Lipid Res. 43 (11), 1960–1968. 10.1194/jlr.m200297-jlr200 12401895

[B35] Wang H. H. Bari O. Arnatt C. K. Liu M. Portincasa P. Wang D. Q. H. (2020a). Activation of Estrogen Receptor G Protein-Coupled Receptor 30 Enhances Cholesterol Cholelithogenesis in Female Mice. Hepatology 72 (6), 2077–2089. 10.1002/hep.31212 32112420 PMC8157628

[B36] Wang Q. Hao C. Yao W. Zhu D. Lu H. Li L. (2020b). Intestinal flora Imbalance Affects Bile Acid Metabolism and Is Associated with Gallstone Formation. BMC Gastroenterol. 20 (1), 59. 10.1186/s12876-020-01195-1 32143645 PMC7060658

[B37] Wang Q. Jiao L. He C. Sun H. Cai Q. Han T. (2017). Alteration of Gut Microbiota in Association with Cholesterol Gallstone Formation in Mice. BMC Gastroenterol. 17 (1), 74. 10.1186/s12876-017-0629-2 28599622 PMC5466737

[B38] Wang T. Y. Portincasa P. Liu M. Tso P. Wang D. Q.-H. (2018). Mouse Models of Gallstone Disease. Curr. Opin. Gastroenterol. 34 (2), 59–70. 10.1097/MOG.0000000000000417 29266008 PMC5938553

[B39] Wu T.-R. Lin C.-S. Chang C.-J. Lin T.-L. Martel J. Ko Y.-F. (2019). Gut Commensal Parabacteroides Goldsteinii Plays a Predominant Role in the Anti-obesity Effects of Polysaccharides Isolated from Hirsutella Sinensis. Gut 68 (2), 248–262. 10.1136/gutjnl-2017-315458 30007918

