# Supplementary figures and images for: Astragalus Polysaccharides Ameliorate Diet-Induced Gallstone Formation by Modulating Synthesis of Bile Acids and the Gut Microbiota
Source: Front Pharmacol. 2021 Jul 1;12:701003. doi: 10.3389/fphar.2021.701003 (PMC8281024; doi:10.3389/fphar.2021.701003)

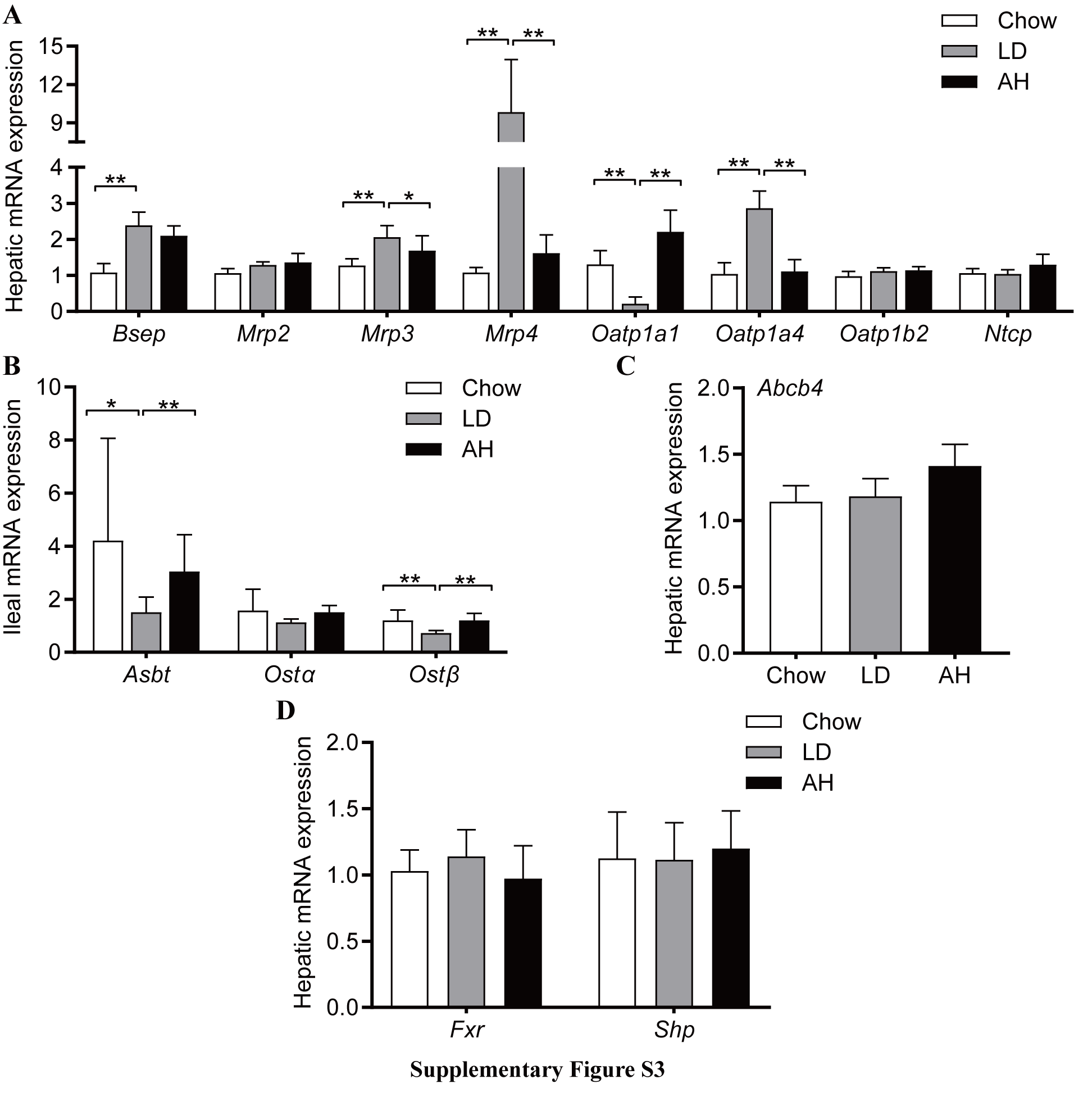

Supplement: Supplementary file 1 [file Image3.TIF]

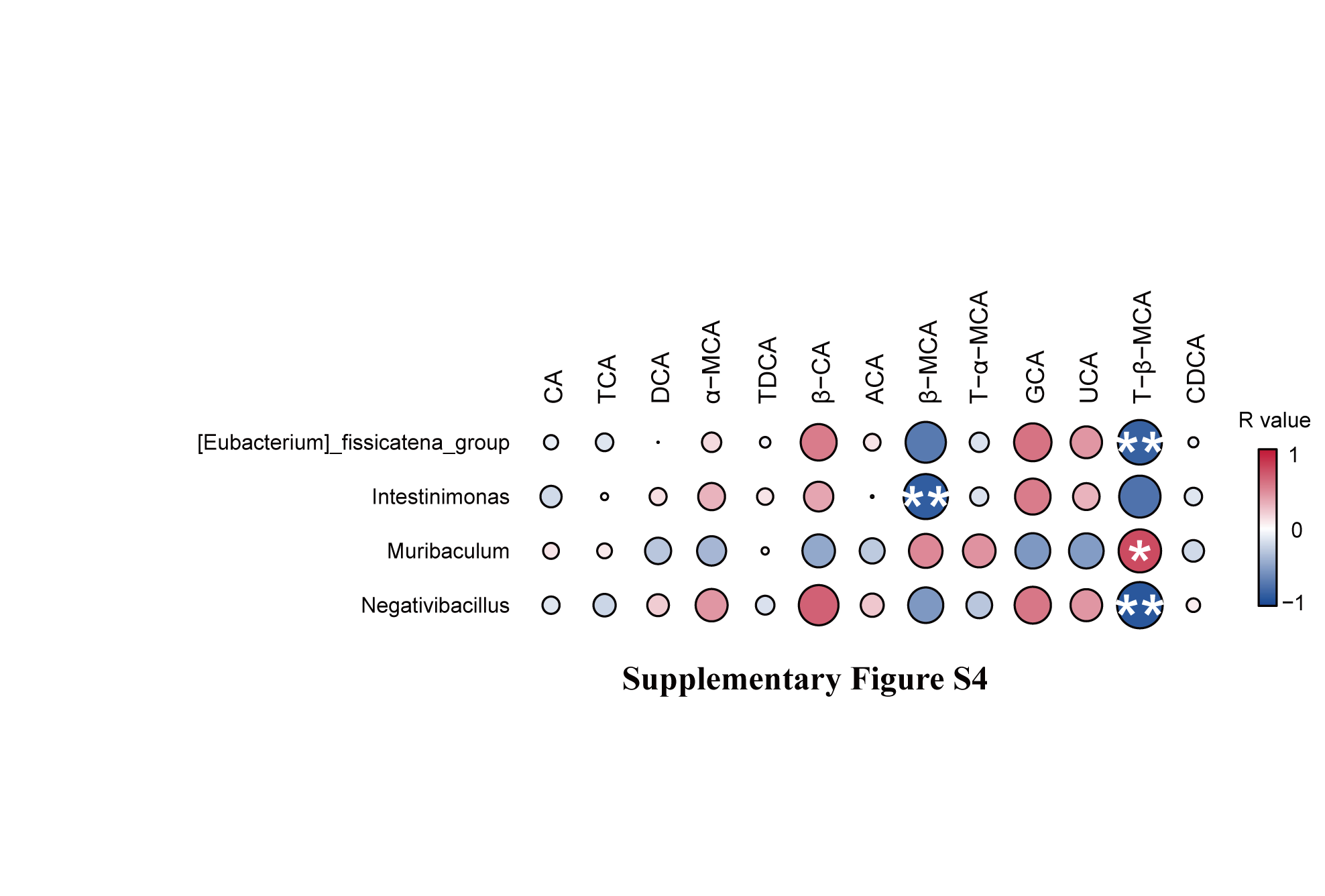

Supplement: Supplementary file 2 [file Image4.TIF]

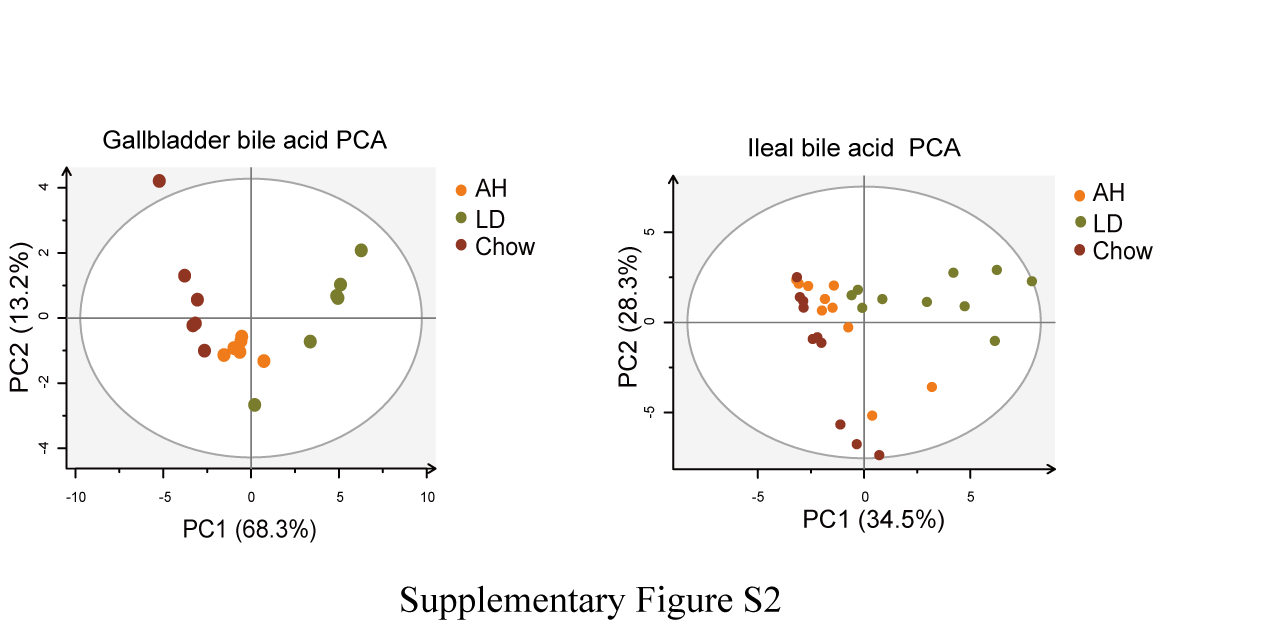

Supplement: Supplementary file 3 [file Image2.TIF]

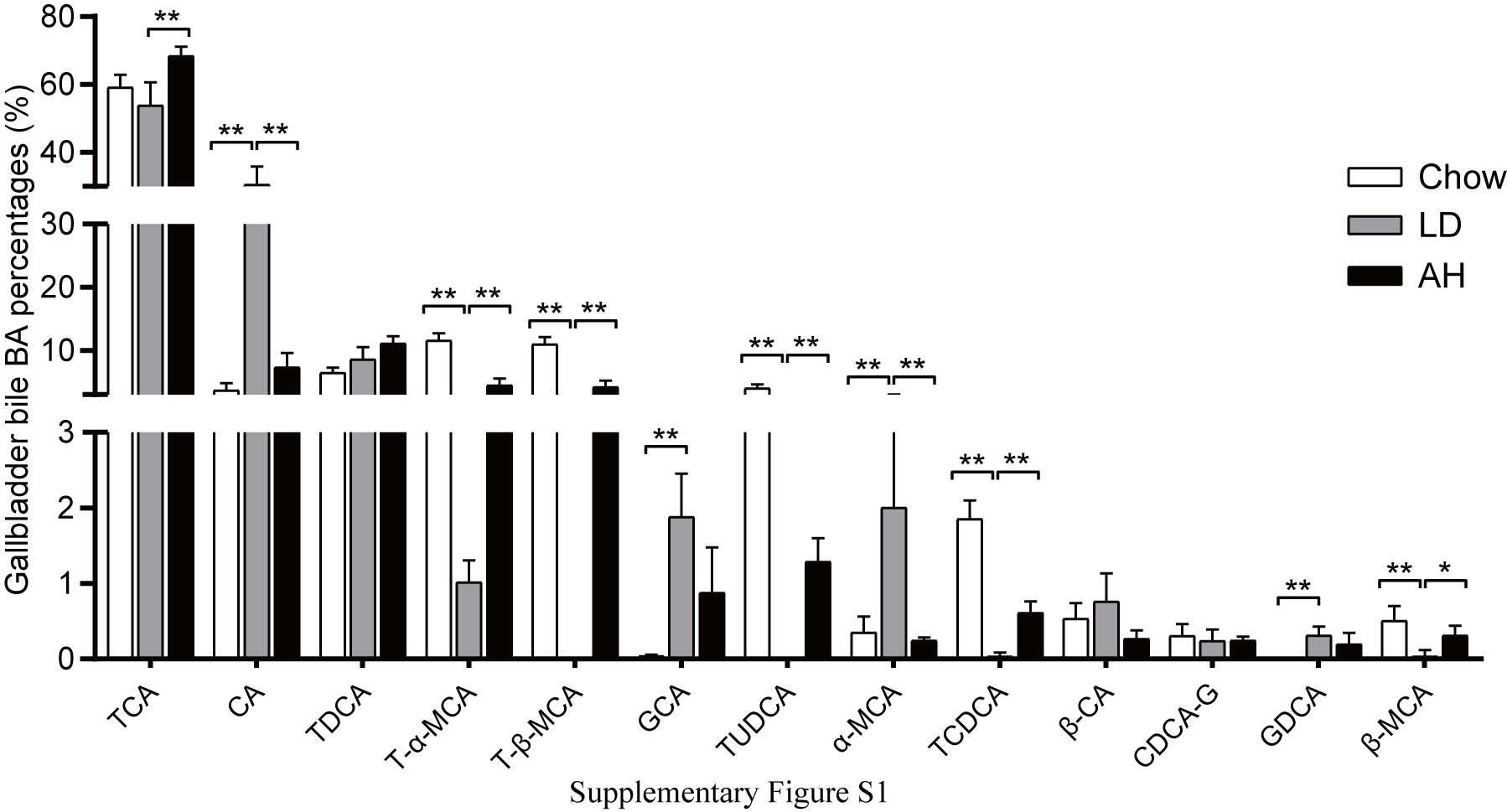

Supplement: Supplementary file 4 [file Image1.TIF]
